# Prognostic utility of neutrophil gelatinase-associated lipocalin (NGAL) levels for cardiovascular events in patients with stable coronary artery disease treated with percutaneous coronary intervention: a prospective longitudinal cohort study

**DOI:** 10.1186/s40364-025-00737-7

**Published:** 2025-02-07

**Authors:** Ting-Yu Lin, Hsin-Bang Leu, Yen-Wen Wu, Wei-Kung Tseng, Tsung-Hsien Lin, Hung-I Yeh, Kuan-Cheng Chang, Ji-Hung Wang, Wei-Hsian Yin, Chau-Chung Wu, Chun-Yao Huang, Shing-Jong Lin, Chien-Yi Hsu, Jaw-Wen Chen

**Affiliations:** 1 School of Medicine, National Yang Ming Chiao Tung University, Taipei, Taiwan; 2 Division of Cardiology, Department of Internal Medicine, Taipei Veterans General Hospital, Taipei, Taiwan; 3 Heath Care and Management Center, Taipei Veterans General Hospital, Taipei, Taiwan; 4 Cardiovascular Medical Center, Kaohsiung Veterans General Hospital, Kaohsiung, Taiwan; 5 Cardiology Division of Cardiovascular Medical Center, Department of Nuclear Medicine, Far Eastern Memorial Hospital, New Taipei City, Taiwan; 6 Division of Cardiology, Department of Internal Medicine, E-Da Hospital, Kaohsiung, Taiwan; 7 Division of Cardiology, Department of Internal Medicine, Faculty of Medicine, Graduate Institute of Medicine, College of Medicine, Kaohsiung Medical University Hospital, Kaohsiung Medical University, Kaohsiung, Taiwan; 8 Division of Cardiology, Department of Internal Medicine, MacKay Memorial Hospital, MacKay Medical College, New Taipei City, Taiwan; 9 Division of Cardiovascular Medicine, Department of Medicine, China Medical University Hospital, Taichung, 404327 Taiwan; 10 Graduate Institute of Biomedical Sciences, Taiwan, China Medical University, Taichung, 404333 Taiwan; 11 Department of Cardiology, Buddhist Tzu-Chi General Hospital, Tzu-Chi University, Hualien, Taiwan; 12 Heart Center, Faculty of Medicine, School of Medicine, Cheng Hsin General Hospital, National Yang Ming Chiao Tung University, Taipei, Taiwan; 13 Cardiology Division, Department of Internal Medicine, Graduate Institute of Medical Education and Bioethics, College of Medicine, National Taiwan University Hospital, National Taiwan University, Taipei, Taiwan; 14 Division of Cardiology and Cardiovascular Research Center, Department of Internal Medicine, Taipei Medical University Hospital, Taipei, Taiwan; 15 Department of Medical Research, Taipei Medical University Hospital, Taipei, Taiwan; 16 Division of Cardiology, Department of Internal Medicine, School of Medicine, College of Medicine, Taipei Medical University, Taipei, Taiwan; 17 Taipei Heart Institute, Taipei Medical University, Taipei, Taiwan

## Abstract

**Introduction:**

Neutrophil gelatinase-associated lipocalin (NGAL) modulates the enzymatic activity of matrix metalloproteinase-9, which is an important mediator of plaque instability in atherosclerosis. High NGAL levels can independently predict all-cause mortality and major adverse cardiac events (MACE) in patients with acute myocardial infarction (AMI). However, studies that have measured NGAL levels in patients with stable coronary artery disease (CAD) are limited. Furthermore, no significant prognostic predictive value between NGAL levels and stable CAD has been established.

**Hypothesis:**

We aimed to investigate the prognostic role of NGAL levels in a prospective cohort study of patients with stable CAD treated with percutaneous coronary intervention (PCI).

**Methods:**

A total of 2,238 stable patients with CAD and a previous PCI were enrolled in a multicenter prospective observational study (The National Taiwan Biosignature Research, NTBR) in Taiwan. The primary outcome was the occurrence of MACE (cardiovascular death, nonfatal myocardial infarction, and ischemic stroke). The secondary outcome was a composite of cardiovascular events (cardiovascular death, nonfatal MI, nonfatal stroke, and hospitalization for heart failure).

**Results:**

During the mean follow-up period of 4.6 ± 1.7 years, 441 patients reached the primary endpoints. Kaplan-Meier analysis showed that event-free survival was significantly different between the first and third tertile groups (log-rank test, *p* < 0.001) in subjects categorized by NGAL levels. In a multivariate Cox proportional hazard regression analysis, plasma NGAL levels were independently associated with an increased risk of MACE [adjusted hazard ratio (aHR) = 1.35; 95% confidence interval (CI) = 1.18–1.54, *p* < 0.001], AMI (aHR = 1.34; 95% CI = 1.12–1.59, *p* < 0.001), and target vessel revascularization (aHR = 1.35; 95% CI = 1.19–1.53, *p* < 0.001). Addition of serum NGAL levels to the traditional risk model improved its prediction value for future cardiovascular events.

**Conclusions:**

High plasma NGAL levels were independently associated with the occurrence of MACE and composite cardiovascular events in patients with stable PCI-treat CAD.

## Introduction

Coronary artery disease (CAD) and acute coronary syndrome (ACS), which are caused by atherosclerosis and plaque destabilization, are the result of vascular inflammation, endothelial dysfunction, and hypercoagulability. Serum biomarker levels reflect the pathophysiological constituents of CAD and thus, may be used for risk stratification, diagnosis, severity evaluation, or prognosis prediction. Several novel biomarkers are being investigated for their potential clinical value in CAD, of which neutrophil gelatinase-associated lipocalin (NGAL) has been promising. NGAL reportedly assists in the early detection of acute kidney injury (AKI) and thus, reduces the morbidity and mortality of patients with cardiorenal syndrome [1]. One study reported that NGAL could predict mortality in patients with heart failure (HF) with or without renal dysfunction [2]. We selected NGAL as a prognostic marker due to its emerging role in cardiovascular (CV) diseases, particularly its association with inflammation and plaque instability, which are critical in the pathogenesis of coronary artery disease (CAD).

NGAL is a 25-kDa matrix metalloproteinase-9 (MMP-9)-bound glycoprotein that was initially isolated from neutrophils [3]. It is found in cardiomyocytes, renal tubular cells, and the endothelial system [4, 5]. NGAL is reportedly a marker for neutrophil activation and is involved in systemic inflammation and endothelial dysfunction, leading to atherosclerotic plaque formation [6–8]. Serum NGAL levels are significantly higher in patients with angiographically confirmed CAD than in controls with normal arteries [8]. NGAL expression reportedly increases in patients with acute myocardial infarction (AMI), serving as a long-term prognostic predictor in patients with ST-segment elevation myocardial infarction (STEMI) or non-STEMI (NSTEMI) [9]. While several biomarkers, including high-sensitivity C-reactive protein (hsCRP), interleukin-6 (IL-6), MMP-9, and tumor necrosis factor-α (TNF-α), indicate inflammation, NGAL possesses distinct properties. Although hsCRP, IL-6, and TNF-α are potent indicators of systemic inflammation, their levels may be elevated in various inflammatory conditions, such as autoimmune diseases, infections, and chronic inflammatory disorders. This lack of specificity limits their reliability in reflecting plaque stability in CAD populations, particularly when compared to MMP-9 and NGAL. While MMP-9 is directly linked to plaque instability, NGAL regulates its effects by binding and stabilizing MMP-9, thereby enhancing its activity. This interaction renders NGAL a more robust marker for CAD prognosis than MMP-9. NGAL has demonstrated predictive value for adverse outcomes in acute myocardial infarction (AMI) and heart failure (HF), suggesting its potential as a valuable biomarker for broader cardiovascular risk stratification. However, the predictive value of NGAL in patients with stable CAD remains controversial [10–12]. Thus, we aimed to assess the predictive value of serum NGAL levels for major adverse CV events (MACE) in patients with stable CAD.

## Methods

### Study population

All patients with stable CAD were recruited from the National Taiwan Biosignature Research (NTBR) cohort study between July 2012 and December 2016. Patients were initially evaluated at one of nine medical centers located in northern, central, southern, and eastern Taiwan [13]. Inflammation-related biomarkers, including NGAL, were estimated at the initial enrollment. Patients were initially evaluated for a history of significant CAD, which was diagnosed if at least one of the following criteria wad fulfilled: (1) ischemic change on a 12-lead electrocardiography (ECG), elevated cardiac enzymes, and a diagnosis of AMI in the medical records; or (2) symptoms of angina with ischemic changes on a 12-lead ECG or a positive stress test. Patients were enrolled if (1) they had a history of at least one previously successful percutaneous coronary intervention (PCI) with either coronary stenting or balloon angioplasty, and (2) they had been stable on medical treatment for at least 1 month before enrollment. Patients were excluded if they: (1) had been hospitalized for any CV event within the last 3 months, (2) they had significant malignancy requiring hospitalization or operation, (3) they had other major systemic diseases requiring hospitalization or operation, (4) life expectancy of < 6 months, (5) they were receiving treatment of immunosuppressive agents, or (6) they were unable or unwilling to be followed up during thefor 1 year. The study was conducted in accordance with the principles of the Declaration of Helsinki and was approved by the Ethics Committees and Independent Review Boards of each hospital in Taiwan.

### Baseline data collection

After enrollment, the following baseline characteristics were collected by physicians and nurses and stored on web-based electronic medical record system at each collaboration center: sex, age, body mass index, hypertension, diabetes, smoking status, number of diseased coronary arteries, family history of CAD, laboratory data including serum creatinine levels, lipid profile, and inflammation-related biomarker levels, and medication underuse.

### Laboratory assessment

After an overnight fast of > 8 h, blood samples were obtained from all patients and stored at − 80 °C for the measurement of biomarker, serum creatinine, and fasting glucose levels, estimated glomerular filtration rate (eGFR), and fasting lipid profile such as triglyceride, total cholesterol, high-density lipoprotein cholesterol (HDL-C), and low-density lipoprotein cholesterol (LDL-C) levels. Serum NGAL levels were measured using standard enzyme-linked immunosorbent assay (ELISA) kits following the manufacturer’s instructions. We used the Merck MILLIPLEX^®^ MAP kit and Human Cardiovascular Disease (HCVD) Panel 2 for quantitative detection of NGAL levels. All reagents were stored at 2–8 °C. Serum samples were diluted 1:100 in the assay buffer provided with the kit, centrifuged to remove debris, and stored at ≤ -20 °C until analysis. Serum levels of N-terminal pro-B-type natriuretic peptide (NT-pro BNP), high-sensitivity C-reactive protein (hsCRP), adiponectin, lipoprotein-associated phospholipase A2 (LpPLa2), tumor necrosis factor-α (TNF-α), interleukin-6 (IL-6), and MMP-9 levels were measured using immunoturbidimetric assays. The serum levels of each biomarker were determined using ELISA kits established in our laboratories [13–15].

### Clinical follow-up and outcomes

The primary outcome of this study was the first occurrence of MACE, which was defined as a composite of CV death, nonfatal MI, and nonfatal stroke. The secondary outcome was a composite of CV events, including CV death, nonfatal MI, nonfatal stroke, and hospitalization for congestive HF (CHF). Individual outcomes, including nonfatal MI, CV death, hospitalization for CHF, and target vessel revascularization were also analyzed. Patients with an initially stable condition under medical treatment were prospectively and regularly followed-up in the outpatient clinic approximately every 3 months for the first year and thereafter, every 6 months. Each patient could contribute one or more events to the analysis. MI was defined as elevated serum cardiac enzyme levels and characteristic ECG changes. Stroke was defined as reduced blood flow to a part of or the whole brain, tissue damage and function loss, and supported by radiological evidence, such as computed tomography or magnetic resonance imaging. CV death is death due to a CV cause such as MI, heart failure, stroke, CV-related hemorrhage, CV procedure-related death, or sudden cardiac death. Target vessel revascularization was defined as any clinically driven repeat PCI or coronary artery bypass grafting of the target vessel. New-onset hemodialysis was defined as the initiation of hemodialysis after the index PCI procedure. All clinical events were confirmed by the Welfare Data Science Center of Taiwan. The study’s last follow-up date on December 31, 2019.

### Statistical analysis

Baseline patient characteristics were compared according to serum NGAL levels. Quantitative variables are expressed as mean and standard deviation in the presence of a normal distribution. Qualitative variables are presented as absolute (number of patients) and relative (percentages) frequencies. The continuous variables were compared using ANOVA, while the categorical variables were compared using χ [2] or Fisher’s exact test. Each outcome is expressed as the number of patients and corresponding percentages. Given that renal dysfunction can independently elevate NGAL levels regardless of cardiovascular disease status, renal function is a critical factor in evaluating the association between NGAL and cardiovascular outcomes. Serum creatinine, a widely recognized marker for renal function, was used to account for the effect of renal impairment on NGAL levels, allowing us to more accurately isolate NGAL’s potential prognostic value beyond its relationship with renal function. Cox proportional hazards regression analysis was performed to estimate hazard ratios (HRs) and 95% confidence intervals (CIs) for each outcome. Three sequential models were constructed to adjust for well-established risk factors for coronary artery disease (CAD) prognosis: (1) age, sex, hypertension, diabetes, and smoking status (Adjusted-1); (2) Adjusted-1 factors plus serum creatinine (Adjusted-2); and (3) Adjusted-2 factors plus serum LDL-C (Adjusted-3). For all analyses, patients in N-GAL tertile 1 (T1) served as the reference group. Additionally, N-GAL was analyzed as a continuous variable, with an increment of N-GAL levels in 500 ng/mL, to further investigate its relationship with outcomes. The Akaike Information Criterion (AIC) was used to objectively evaluate and compare the quality of these models, with lower AIC values indicating better model performance. Event-free survival curves of patients stratified by serum NGAL tertiles were generated using the Kaplan–Meier method and evaluated for significance with log-rank tests. To assess whether the prediction of MACE would improve after the addition of NGAL to a baseline model with traditional and well-established risk factors (i.e., age, sex, hypertension, diabetes, smoking status, serum creatinine and serum LDL-C level), the net reclassification improvement (NRI), integrated discrimination improvement (IDI), receiver operating characteristic (ROC) analysis and calculated the area under the curve (AUC) were calculated. For all tests, the two-tailed α significance level was set at 0.05.

## Results

### Baseline patient characteristics

A total of 2,238 patients with stable CAD and previous PCI were enrolled in the study. The mean follow-up duration was 4.6 ± 1.7 years. The clinical characteristics of the patients stratified by serum NGAL tertiles are summarized in Table 1. The mean age was similar among the three groups (62–64 years). The study population was male predominant, accounting for 80–90% of all three groups. There were 1429 (64.9%) patients with hypertension, 796 (36.1%) with diabetes mellitus, 64 (2.9%) with ischemic stroke, and 435 (19.7%) with a family history of CAD. There were no significant differences in the prevalence of underlying comorbidities or target lesion characteristics among patients stratified by NGAL tertiles, except in the single-vessel disease (SVD) category. While previous studies have demonstrated a correlation between NGAL levels and CAD severity [8, 16], our study did not find this association. One possible explanation is that categorizing vessel involvement as single, double, or triple-vessel disease (SVD, DVD, TVD) may not fully represent CAD severity compared to a more detailed measure like the SYNTAX score. Consequently, even within the same category of vessel involvement, variations in SYNTAX scores may still influence serum NGAL levels. Given this limitation, we should interpret the significance of this finding with caution. In the baseline laboratory data, some biomarkers were significantly different between NGAL tertiles and showed some correlation. In patients with higher serum NGAL levels, the left ventricular ejection fraction (*p* = 0.002), hemoglobin levels (*p* = 0.015), and eGFR (*p* < 0.0001) were lower and the fasting plasma glucose (*p* < 0.0001), serum creatinine (*p* < 0.0001), and NT-pro BNP (*p* < 0.0001) levels were higher. Patients with higher tertiles of serum NGAL levels had significantly higher levels of hsCRP (*p* = 0.001), IL-6 (*p* = 0.001), TNF-(*p* < 0.0001), and MMP-9 (*p* < 0.0001). There was no significant difference in adiponectin or LppPLa2 levels between NGAL tertiles. There was no significant difference in the lipid profile, which included total cholesterol and LDL-C levels, and medication use, including that of oral antiplatelets, beta-blockers, dihydropyridine calcium channel blockers, diuretics, and statins, between the NGAL tertiles.

**Table 1:** Baseline characteristics of the patients stratified by serum NGAL tertiles

Characteristics	Tertiles of NGAL				*p*-value
	T1 (0.3–178 ng/ml) *N* = 734	T2 (178.22–333.86 ng/ml) *N* = 734	T3 (334.57–1950 ng/ml) *N* = 733		
**Basic profiles**					
Age, y	63.22 ± 10.27	62.52 ± 12.30	63.57 ± 12.46	0.216	
Men, n (%)	605 (82)	616 (84)	640 (87)	0.030	
BMI, kg/m^2^	26.46 ± 4.01	26.68 ± 4.63	26.36 ± 4.22	0.348	
Waist circumference, cm	92.02 ± 9.95	92.89 ± 10.12	93.59 ± 10.23	0.012	
Buttock circumference, cm	98.49 ± 8.55	98.89 ± 8.74	98.94 ± 9.11	0.578	
Waist-buttock ratio	0.93 ± 0.06	0.94 ± 0.08	0.95 ± 0.06	0.005	
Systolic BP, mm Hg	129.94 ± 17.35	129.99 ± 16.98	131.51 ± 19.52	0.165	
Diastolic BP, mm Hg	75.20 ± 11.48	74.56 ± 11.73	75.22 ± 12.62	0.487	
Family history of CAD, n (%)	137 (19)	161 (22)	137 (19)	0.195	
Smokers, n (%)	367 (50)	378 (52)	439 (60)	< 0.0001	
**Comorbidities**					
Hypertension, n (%)	495 (67)	479 (65)	455 (62)	0.096	
Diabetes mellitus, n (%)	257 (35)	259 (35)	280 (38)	0.371	
Ischemic stroke, n (%)	15 (2.04)	22 (3)	27 (3.68)	0.172	
**Cardiovascular profiles**					
LVEF (%)	59.22 ± 13.32	60.50 ± 12.54	57.61 ± 12.48	0.002	
**Target lesions**					
Left main, n (%)	14 (1.91)	12 (1.63)	17 (2.32)	0.762	
LAD, n (%)	248 (33.79)	240 (32.7)	258 (35.2)	0.069	
LCX, n (%)	112 (15.26)	126 (17.17)	130 (17.74)	0.842	
RCA, n (%)	145 (19.75)	164 (22.34)	190 (25.92)	0.617	
SVD, n (%)	330 (44.96)	330 (44.96)	352 (48.02)	0.038	
DVD, n (%)	83 (11.31)	95 (12.94)	99 (13.51)	0.902	
TVD, n (%)	7 (0.95)	8 (1.09)	14 (1.91)	0.409	
**Baseline laboratory data**					
Hemoglobin, g/dL	13.78 ± 1.69	13.76 ± 1.81	13.52 ± 2.07	0.015	
FPG, mg/dL	116.13 ± 37.41	120.53 ± 42.67	125.66 ± 49.70	< 0.0001	
Serum creatinine, mg/dL	1.02 ± 0.47	1.14 ± 0.59	1.70 ± 2.07	< 0.0001	
eGFR, mL/min per 1.73 m^2^	82.05 ± 24.63	77.26 ± 29.40	70.74 ± 33.89	< 0.0001	
NT-pro BNP, pg/mL	247 ± 351	237 ± 336	358 ± 422	< 0.0001	
**Lipid profile**					
Total cholesterol, mg/dL	160.26 ± 35.27	163.76 ± 37.10	162.16 ± 38.67	0.197	
Triglyceride, mg/dL	134.15 ± 92.84	140.02 ± 85.34	142.09 ± 100.84	0.241	
HDL-C, mg/dL	43.08 ± 11.30	42.69 ± 11.26	40.44 ± 10.40	< 0.0001	
LDL-C, mg/dL	92.45 ± 27.91	95.48 ± 31.41	95.11 ± 31.98	0.117	
Total cholesterol/ HDL-c ratio	3.93 ± 1.21	4.04 ± 1.21	4.22 ± 1.35	< 0.0001	
**Inflammation-related biomarkers**					
HsCRP, mg/dL	0.19 ± 0.26	0.23 ± 0.33	0.28 ± 0.35	0.001	
Adiponectin, ng/mL	6,851 ± 5,341	6,297 ± 4,280	7,148 ± 5,112	0.073	
LppPLa2, ng/mL	117 ± 147	134 ± 162	148 ± 186	0.052	
IL-6, pg/mL	2.34 ± 2.24	2.37 ± 2.11	2.94 ± 2.48	0.001	
TNF-α, pg/mL	2.58 ± 1.59	2.75 ± 1.58	3.81 ± 2.54	< 0.0001	
MMP-9, pg/mL	377.62 ± 313.43	616.43 ± 393.18	648.83 ± 392.23	< 0.0001	
**Medications**					
Oral antiplatelets, n (%)	683 (93)	682 (93)	687 (94)	0.804	
Oral anricoagulants, n (%)	21 (2.86)	16 (2.18)	22 (3)	0.581	
ACEI inhibitors, n (%)	124 (17)	155 (21)	173 (24)	0.006	
ARB, n (%)	332 (45)	330 (45)	290 (40)	0.047	
beta-blockers, n (%)	501 (68)	494 (67)	462 (63)	0.079	
DHP-CCB, n (%)	254 (35)	247 (34)	214 (29)	0.062	
Diuretics, n (%)	99 (13.49)	97 (13.22)	93 (12.69)	0.899	
Nitrates, n (%)	284 (39)	345 (47)	339 (46)	0.002	
Statins, n (%)	539 (73)	569 (78)	555 (76)	0.189	

Data are presented as means ± standard deviation. ACE, angiotensin-converting enzyme; ARB, angiotensin receptor blocker; BMI, body mass index; BP, blood pressure; CAD, coronary artery disease; DHP-CCB, dihydropyridine calcium channel blocker; eGFR, estimated glomerular filtration rate; FPG, fasting plasma glucose; HDL, high-density lipoprotein; hsCRP, high-sensitivity C-reactive protein; IL-6, interleukin-6; LAD, left anterior descending artery; LCX, left circumflex artery; LDL, low-density lipoprotein; LpPLa2, lipoprotein-associated phospholipase A2; LVEF, left ventricular ejection fraction; MMP-9, matrix metallopeptidase-9; NT-pro BNP, N-terminal pro-B-type natriuretic peptide; RCA, right coronary artery; TNF-α, tumor necrosis factor-α; TNFSF14, tumor necrosis factor superfamily 14; WBC, white blood count, SVD, single vessel disease; DVD, double vessel disease; TVD, triple vessel disease

### Association between serum NGAL levels and CV outcomes in patients after PCI

Among the 2,238 enrolled patients with CAD, seven outcomes were identified, including 255 (11.4%) AMIs, 163 (7.3%) CV deaths, 288 (12.9%) CHFs, 441 (19.7%) MACEs, 579 (25.9%) composite of CV events, 512 (22.9%) target vessel revascularizations and 82 (3.7%) new-onset hemodialysis. Patient with higher serum NGAL levels (higher tertiles) had a higher risk of AMI (*p* = 0.001), CV death (*p* < 0.0001), CHF (*p* < 0.0001), MACE (*p* < 0.0001), composite of CV events (*p* < 0.0001), target vessel revascularization (*p* < 0.0001) and new-onset hemodialysis (*p* < 0.001) (Table 2).

**Table 2:** Clinical outcomes of patients stratified by serum NGAL tertiles

Outcomes	Level of *N*-GAL				*P*-value
	Overall (*n* = 2238)	T1(0.3 ∼ 178 ng/ml) (*n* = 734)	T2(178.22 ∼ 333.86 ng/ml) (*n* = 734)	T3(334.57 ∼ 1950 ng/ml) (*n* = 733)	
AMI,	255	62 (24.3)	84 (32.9)	109 (42.7)	0.001
CV death,	163	45 (27.6)	39 (23.9)	79 (48.5)	< 0.0001
CHF	306	78 (27.1)	97 (33.7)	131 (45.5)	< 0.0001
MACE	441	119 (27.0)	138 (31.3)	184 (41.7)	< 0.0001
A composite of CV events*	579	156 (27.0)	190 (32.8)	233 (40.2)	< 0.0001
Revascularization	512	137 (26.8)	165 (32.2)	210 (41.0)	< 0.0001
New-onset Hemodialysis	82	11(13.4)	28(34.1)	43(52.4)	< 0.0001

Data are presented as n (%). AMI, acute myocardial infarction; CV, cardiovascular; CHF, congestive heart failure; MACE, major adverse cardiovascular events

*: includes MACE and CHF

In patients with serum NGAL levels in the highest tertile (T3), the adjusted HRs (adjusted-1) were significantly higher compared to those in the lowest tertile (T1) for various outcomes: AMI (HR = 1.81, 95% CI = 1.32–2.47, *p* < 0.001), CV death (HR = 1.73, 95% CI = 1.20–2.51, *p* = 0.004), CHF (HR = 1.75, 95% CI = 1.32–2.32, *p* < 0.001), MACE (HR = 1.58, 95% CI = 1.25–1.99, *p* < 0.001), composite CV events (HR = 1.57, 95% CI = 1.28–1.93, *p* < 0.001), target vessel revascularization (HR = 1.66, 95% CI = 1.34–2.06, *p* < 0.001), and new-onset hemodialysis (HR = 4.17, 95% CI = 2.16–8.11, *p* < 0.001). The p-trend across NGAL tertiles was significant for AMI (p-trend < 0.001), CV death (*p* = 0.002), CHF (p-trend < 0.001), MACE (p-trend < 0.001), composite CV events (p-trend < 0.001), target vessel revascularization (p-trend < 0.001), and new-onset hemodialysis (p-trend < 0.001).

After further adjustment for serum creatinine levels (adjusted-2), the HRs remained significantly elevated for AMI (HR = 1.57, 95% CI = 1.14–2.17, *p* = 0.006), CHF (HR = 1.48, 95% CI = 1.11–1.97, *p* = 0.008), MACE (HR = 1.34, 95% CI = 1.06–1.71, *p* = 0.016), composite CV events (HR = 1.34, 95% CI = 1.09–1.66, *p* = 0.006), target vessel revascularization (HR = 1.50, 95% CI = 1.20–1.87, *p* < 0.001), and new-onset hemodialysis (HR = 2.95, 95% CI = 1.49–5.86, *p* = 0.002). However, the significance for CV death was no longer observed.

After additional adjustment for serum LDL-C levels (adjusted-3), HRs for AMI (HR = 1.52, 95% CI = 1.10–2.10, *p* = 0.011), CHF (HR = 1.45, 95% CI = 1.08–1.95, *p* = 0.012), MACE (HR = 1.31, 95% CI = 1.03–1.66, *p* = 0.030), composite CV events (HR = 1.32, 95% CI = 1.07–1.63, *p* = 0.010), target vessel revascularization (HR = 1.47, 95% CI = 1.17–1.84, *p* = 0.001), and new-onset hemodialysis (HR = 2.91, 95% CI = 1.46–5.79, *p* = 0.002) remained significantly elevated in T3 compared to T1.

The AIC was used to evaluate model quality when patients were stratified by N-GAL tertiles. The Adjusted-3 model consistently demonstrated the lowest AIC values across outcomes, including AMI, CV death, CHF, MACE, composite CV events, and revascularization, indicating the best balance of fit and complexity.

Analyzing NGAL levels as a continuous variable in the adjusted-1 model revealed higher serum NGAL levels were significantly associated with increased risks of AMI (HR = 1.34, 95% CI = 1.12–1.59, *p* = 0.001), CV death (HR = 1.60, 95% CI = 1.32–1.95, *p* < 0.001), CHF (HR = 1.37, 95% CI = 1.17–1.61, *p* < 0.001), MACE (HR = 1.35, 95% CI = 1.18–1.54, *p* < 0.001), composite CV events (HR = 1.31, 95% CI = 1.17–1.48, *p* < 0.001), target vessel revascularization (HR = 1.35, 95% CI = 1.19–1.53, *p* < 0.001), and new-onset hemodialysis (HR = 1.47, 95% CI = 1.11–1.95, *p* = 0.007).

After adjusting for serum creatinine levels in the adjusted-2 model, higher NGAL levels were still associated with increased risks of CV death (HR = 1.30, 95% CI = 1.04–1.16, *p* = 0.021), MACE (HR = 1.17, 95% CI = 1.01–1.35, *p* = 0.039), composite CV events (HR = 1.15, 95% CI = 1.01–1.31, *p* = 0.042), and target vessel revascularization (HR = 1.23, 95% CI = 1.07–1.41, *p* = 0.003). However, the associations with AMI, CHF, and new-onset hemodialysis were no longer significant.

In the adjusted-3 model, after further adjustment for serum LDL-C levels, higher NGAL levels remained significantly associated with increased risks of CV death (HR = 1.28, 95% CI = 1.03–1.60, *p* = 0.029), MACE (HR = 1.16, 95% CI = 1.00–1.34, *p* = 0.048), and target vessel revascularization (HR = 1.23, 95% CI = 1.07–1.41, *p* = 0.003). However, the significance for composite CV events was no longer observed (Table 3).

**Table 3:** Cox regression analysis of outcomes in patients stratified by serum NGAL tertiles

Outcome	*N*-GAL level	Number of patients with event(%)	Crude HR				Adjusted-1 h*				Adjusted-2 h**				Adjusted-3 h***			
			HR (95% CI)	*P*-value	*P* trend	AIC	HR (95% CI)	*P*-value	*P* trend	AIC	HR (95% CI)	*P*-value	*P* trend	AIC	HR (95% CI)	*P*-value	*P* trend	AIC
**AMI**					< 0.001	3816			< 0.001	3810			0.006	3772			0.011	3725
	T1	62 (24.3)	1.00( Reference )	-			1.00( Reference )	-			1.00( Reference )	-			1.00( Reference )	-		
	T2	84 (32.9)	1.38(1.00–1.92)	0.052			1.38(0.99–1.91)	0.057			1.34(0.97–1.86)	0.080			1.29(0.93–1.80)	0.126		
	T3	109 (42.7)	1.87(1.37–2.55)	< 0.001			1.81(1.32–2.47)	< 0.001			1.57(1.14–2.17)	0.006			1.52(1.10–2.10)	0.011		
As Continuous variable***		255	1.33(1.12–1.58)	0.001		3821	1.34(1.12–1.59)	0.001		3813	1.16(0.95–1.41)	0.143		3776	1.16(0.95–1.42)	0.142		3727
**CV death**					0.262	2415			0.002	2343			0.090	2286			0.142	2245
	T1	45 (27.6)	1.00( Reference )	-			1.00( Reference )	-			1.00( Reference )	-			1.00( Reference )	-		
	T2	39 (23.9)	0.88(0.57–1.35)	0.554			0.85(0.55–1.31)	0.463			0.85(0.56–1.31)	0.473			0.85(0.56–1.31)	0.470		
	T3	79 (48.5)	1.84(1.28–2.66)	0.001			1.73(1.20–2.51)	0.004			1.36(0.93–2.00)	0.112			1.31(0.89–1.93)	0.167		
As Continuous variable		163	1.59(1.32–1.92)	< 0.001		2413	1.60(1.32–1.95)	< 0.001		2338	1.30(1.04–1.61)	0.021		2284	1.28(1.03–1.60)	0.029		2243
**CHF**					< 0.001	4548			< 0.001	4445			0.008	4379			0.012	4309
	T1	78 (27.1)	1.00( Reference )	-			1.00( Reference )	-			1.00( Reference )	-			1.00( Reference )	-		
	T2	97 (33.7)	1.28(0.95–1.72)	0.107			1.27(0.94–1.71)	0.122			1.26(0.93–1.70)	0.132			1.25(0.92–1.69)	0.146		
	T3	131 (45.5)	1.83(1.38–2.42)	< 0.001			1.75(1.32–2.32)	< 0.001			1.48(1.11–1.97)	0.008			1.45(1.08–1.95)	0.012		
As Continuous variable		306	1.37(1.18–1.61)	< 0.001		4552	1.37(1.17–1.61)	< 0.001		4446	1.18(0.99–1.41)	0.067		4381	1.18(0.99–1.41)	0.068		4310
**MACE**					< 0.001	6549			< 0.001	6500			0.016	4601			0.029	6340
	T1	119 (27.0)	1.00( Reference )	-			1.00( Reference )	-			1.00( Reference )	-			1.00( Reference )	-		
	T2	138 (31.3)	1.20(0.94–1.53)	0.153			1.19(0.93–1.52)	0.165			1.16(0.91–1.48)	0.241			1.14(0.89–1.46)	0.307		
	T3	184 (41.7)	1.66(1.32–2.09)	< 0.001			1.58(1.25–1.99)	< 0.001			1.34(1.06–1.71)	0.016			1.31(1.03–1.66)	0.030		
As Continuous variable		441	1.36(1.20–1.55)	< 0.001		6548	1.35(1.18–1.54)	< 0.001		6496	1.17(1.01–1.35)	0.039		6401	1.16(1.00–1.34)	0.048		6339
Composite CV events****					0.498	8579			< 0.001	8491			0.006	8367			0.011	8276
	T1	156 (27.0)	1.00( Reference )	-			1.00( Reference )	-			1.00( Reference )	-			1.00( Reference )	-		
	T2	190 (32.8)	1.27(1.03–1.57)	0.026			1.27(1.03–1.57)	0.027			1.25(1.01–1.55)	0.037			1.24(1.01–1.54)	0.045		
	T3	233 (40.2)	1.64(1.34–2.01)	< 0.001			1.57(1.28–1.93)	< 0.001			1.34(1.09–1.66)	0.006			1.32(1.07–1.63)	0.010		
As Continuous variable		579	1.32(1.18–1.49)	< 0.001		8581	1.31(1.17–1.48)	< 0.001		8491	1.15(1.01–1.31)	0.042		8369	1.14(1.00–1.30)	0.054		8278
**Revascularization**					0.547	7574			< 0.001	7556			< 0.001	7486			0.001	7432
	T1	137 (26.8)	1.00( Reference )	-			1.00( Reference )	-			1.00( Reference )	-			1.00( Reference )	-		
	T2	165 (32.2)	1.25(1.00–1.57)	0.050			1.23(0.98–1.54)	0.076			1.20(0.96–1.51)	0.109			1.18(0.94–1.48)	0.166		
	T3	210 (41.0)	1.72(1.38–2.13)	< 0.001			1.66(1.34–2.06)	< 0.001			1.50(1.20–1.87)	< 0.001			1.47(1.17–1.84)	0.001		
As Continuous variable		512	1.35(1.19–1.52)	< 0.001		7577	1.35(1.19–1.53)	< 0.001		7557	1.23(1.07–1.41)	0.003		7489	1.23(1.07–1.41)	0.003		7434
**New-onset Hemodialysis**					< 0.001	1210			< 0.001	1168			0.002	1123			0.003	1124
	T1	11(13.4)	1.00( Reference )	-			1.00( Reference )	-			1.00( Reference )	-			1.00( Reference )	-		
	T2	28(34.1)	2.62(1.30–5.26)	0.007			2.60(1.29–5.23)	0.007			2.67(1.33–5.38)	0.006			2.63(1.30–5.29)	0.007		
	T3	43(52.4)	4.18(2.16–8.11)	0.000			4.29(2.20–8.34)	0.000			2.95(1.49–5.86)	0.002			2.91(1.46–5.79)	0.002		
As Continuous variable		82	1.47(1.11–1.95)	0.007		1225	1.60(1.20–2.13)	0.001		1180	1.05(0.73–1.49)	0.800		1134	1.04(0.73–1.48)	0.845		1134

AIC: The Akaike Information Criterion; AMI indicates acute myocardial infarction; CV, cardiovascular; CHF, congestive heart failure; HR: Hazard ratio; and MACE, major adverse cardiovascular event

* Multivariate Cox proportional hazards analysis adjusted for age, sex, HTN, DM and smoking status

** Multivariate Cox proportional hazards analysis adjusted for age, sex, HTN, DM, smoking status, serum creatinine level

*** Multivariate Cox proportional hazards analysis adjusted for age, sex, HTN, DM, smoking status, serum creatinine level and LDL-C

***As continue variable means increment of N-GAL level in Δ500ng/ml

****include MACE and CHF

AIC was also applied to models where N-GAL levels were analyzed as a continuous variable. The Adjusted-3 model demonstrated the lowest AIC values across outcomes, including AMI, CV death, CHF, MACE, composite CV events, revascularization, and new-onset hemodialysis, confirming it as the most statistically robust model.

**Fig. 1:**
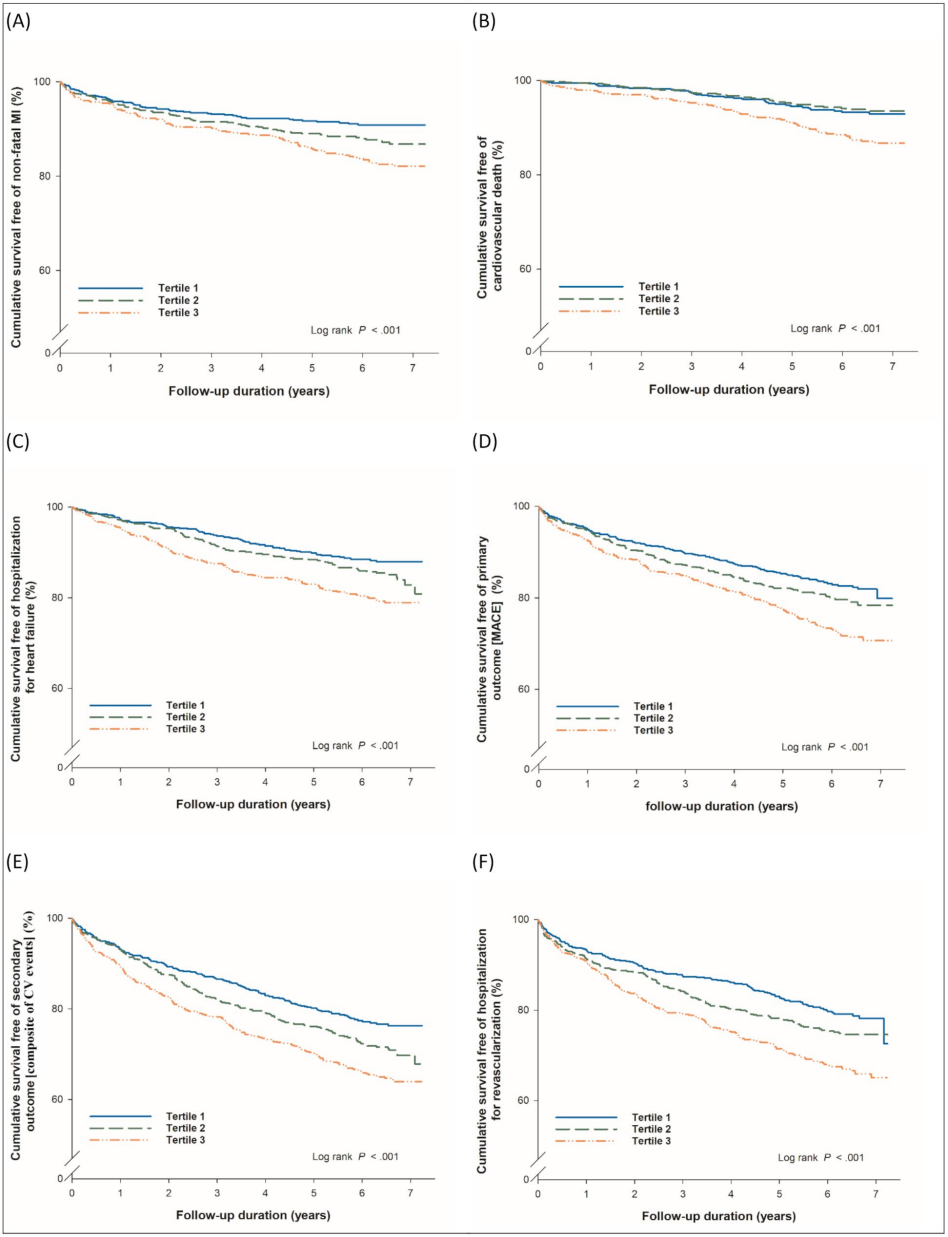
Kaplan-Meier analysis of event-free survival curves for (**A**) myocardial infarction (MI), (**B**) cardiovascular (CV) death, (**C**) heart failure, (**D**) major adverse cardiovascular events (MACE), (**E**) composite of CV events and (**F**) revascularization, in patients stratified by NGAL tertiles

Kaplan-Meier analysis was used for each study outcome in patients stratified by serum NGAL levels. The event-free curves were separated according to T1, T2, and T3 for the outcomes of AMI (log-rank *p* = 0.0003), CV death (log-rank *p* < 0.0001), CHF (log-rank *p* < 0.0001), MACE (log-rank *p* < 0.0001), composite of CV events (log-rank *p* < 0.0001), target vessel revascularization (log-rank *p* < 0.0001), and new-onset hemodialysis (log-rank *p* < 0.0001) (Fig. 1).

### Additional predictive value of NGAL level for future risk of CV events in post-PCI patient with CAD

Adding the serum NGAL levels to a traditional risk model improved the prediction value for AMI (HR = 0.157, 95% CI = 0.0308–0.2831, *p* = 0.019), CV death (HR = 0.230, 95% CI = 0.0736–0.3866, *p* = 0.005), CHF (HR = 0.145, 95% CI = 0.0281–0.2612, *p* = 0.020), MACE (HR = 0.182, 95% CI = 0.0812–0.282, *p* = 0.001), composite of CV events (HR = 0.145, 95% CI = 0.0543 ∼ 0.2355, *p* = 0.003), target vessel revascularization (HR = 0.205, 95% CI = 0.1094–0.2996, *p* < 0.001) and new-onset hemodialysis (HR = 0.276, 95% CI = 0.0592–0.4925, *p* < 0.014), as shown by the significant NRI value. The IDI calculation also revealed similar statistical significance. In the AUC analysis, the inclusion of N-GAL levels in the traditional risk prediction model for AMI and CV death increased the AUC but did not demonstrate statistically significant improvements. However, for CHF, the AUC increased from 0.686 to 0.699 (*P* = 0.017); for total cardiovascular events, the AUC increased from 0.644 to 0.655 (*P* = 0.027); for revascularization, the AUC increased from 0.594 to 0.612 (*P* = 0.028); and for new-onset hemodialysis, the AUC increased from 0.717 to 0.734 (*P* = 0.040) (Table 4).

**Table 4:** Improvement in risk prediction of cardiovascular events in the multivariate-adjusted model after including N-GAL

Outcome	NRI (95% CI)	*P*-value	IDI (95% CI)	*P*-value	AUC	*P*-value
**AMI**						0.279
Traditional risks (Age, Gender, HTN, DM, smoking status, creatinine, LDL)	1.00( Reference )	-	1.00( Reference )	-	0.595	
Traditional risks + N-GAL	0.157(0.0308 ∼ 0.2831)	0.019	0.005(0.0011 ∼ 0.008)	0.009	0.605	
**CV death**						0.113
Traditional risks (Age, Gender, HTN, DM, smoking status, creatinine, LDL)	1.00( Reference )	-	1.00( Reference )	-	0.706	
Traditional risks + N-GAL	0.230(0.0736 ∼ 0.3866)	0.005	0.013(0.0048 ∼ 0.0222)	0.003	0.720	
**CHF**						0.017
Traditional risks (Age, Gender, HTN, DM, smoking status, creatinine, LDL)	1.00( Reference )	-	1.00( Reference )	-	0.686	
Traditional risks + N-GAL	0.145(0.0281 ∼ 0.2612)	0.020	0.005(0.0009 ∼ 0.0091)	0.016	0.699	
**MACE**						0.025
Traditional risks (Age, Gender, HTN, DM, smoking status, creatinine, LDL)	1.00( Reference )	-	1.00( Reference )	-	0.625	
Traditional risks + N-GAL	0.182(0.0812 ∼ 0.282)	0.001	0.009(0.0039 ∼ 0.0137)	0.001	0.641	
**Total CV events**						0.027
Traditional risks (Age, Gender, HTN, DM, smoking status, creatinine, LDL)	1.00( Reference )	-	1.00( Reference )	-	0.644	
Traditional risks + N-GAL	0.145(0.0543 ∼ 0.2355)	0.003	0.008(0.0035 ∼ 0.0119)	< 0.001	0.655	
**Revascularization**						0.028
Traditional risks (Age, Gender, HTN, DM, smoking status, creatinine, LDL)	1.00( Reference )	-	1.00( Reference )	-	0.594	
Traditional risks + N-GAL	0.205(0.1094 ∼ 0.2996)	< 0.001	0.008(0.0041 ∼ 0.0128)	< 0.001	0.612	
**New-onset Hemodialysis**						0.040
Traditional risks (Age, Gender, HTN, DM, smoking status, creatinine, LDL)	1.00( Reference )	-	1.00( Reference )	-	0.717	
Traditional risks + N-GAL	0.276(0.0592 ∼ 0.4925)	0.014	0.004(0.0006 ∼ 0.0078)	0.022	0.734	

NRI, net reclassification improvement; IDI, integrated discrimination improvement; AUC, area under the curve

## Discussion

In our study, baseline serum NGAL levels had the potential to predict MACE independent of age, sex, hypertension, diabetes, smoking status, serum creatinine levels and serum LDL-C levels in stable patients with CAD after a PCI. Compared to patients in the T1 group (with lower serum NGAL levels), patients in the T3 group were associated with a 34% increased risk of MACE and 34% increased risk of a composite of CV events during the follow-up period after complete and adequate adjustment. In Table 3, we observe that while NGAL levels consistently predict other cardiovascular outcomes, the association with CV death appears less linear, particularly in the T2 group, where the HR is slightly below 1 even after adjustment. This unique pattern may be due to the small number of events and potential type II error. Although the HR of CV death in T2 group tends to have HR < 1, the *P* value didn’t reach significance statistically, which explains the lack of a marked difference between T1 and T2 in the Kaplan-Meier curves for CV death. Therefore, the HR < 1 should not be interpreted as indicating a lower risk of CV death in the T2 group compared to T1. Additionally, we conducted a separate Cox regression analysis using NGAL levels categorized in 500 ng/mL increments, which revealed that higher serum NGAL levels is associated with a higher risk of MACE, composite of CV events, CV death, and target vessel revascularization. This approach allowed us to assess risk across a continuous spectrum of NGAL levels, providing additional insight beyond the tertile-based analysis. After adjusting for NGAL levels in the traditional risk model, the positive results of NRI and IDI indicated an increase in its predictive value for clinical outcomes, such as AMI, CV death, CHF, MACE, composite of CV events, and target vessel revascularization. Kaplan-Meier curves revealed that patients with higher serum NGAL levels had lower event-free survival.

Besides, patients in the highest NGAL tertile demonstrated a significantly elevated hazard ratio (HR) for new-onset hemodialysis compared to those in the lowest tertile, with a clear p-trend across tertiles. Furthermore, incorporating NGAL levels into a model with traditional risk factors enhanced the predictive performance for new-onset hemodialysis. NGAL has been established as a biomarker for predicting long-term renal outcomes in patients with heart failure and those undergoing cardiac surgery [17, 18]. The alignment of our findings with these studies suggests that NGAL’s prognostic utility extends to identifying the risk of chronic renal deterioration requiring dialysis, even within a stable CAD population.

NGAL is reportedly a biomarker of renal injury and has been used clinically as an accurate parameter for estimating renal functions [19]. NGAL also contributes significantly to the inflammatory processes involving atherosclerosis or plaque instability [20]. NGAL is a glycoprotein stored in the granules of mature neutrophils [3], which plays an important role in the progression of atherosclerosis [21]. Recently, increased NGAL expression has been detected in cardiomyocytes and macrophages in atherosclerotic plaques; thus, epithelial damage can significantly elevate serum NGAL levels [7, 22]. Boekhorst et al. determined that NGAL is highly expressed in human atherosclerotic lesions and is associated with increased MMP-9 activity [23]. MMP-9 is an endopeptidase expressed in vulnerable atherosclerotic plaques, which causes collagen degradation with subsequent central necrosis, intramural hematoma, or plaque rupture. The main mechanism responsible for the inactivation of MMP-9 is inhibition by tissue inhibitor of metalloproteinase (TIMP-1), and the imbalance between this inhibition and the proteolysis by MMP-9 will lead to plaque rupture and subsequent ACS [24]. NGAL can form a stable, biologically active complex with MMP-9 and prevent inactivation by TMP-1, leading to enhanced proteolytic activity and plaque instability [25]. In the current study, the blood concentrations of NGAL and MMP-9 in our CAD cohort showed a positive correlation (Table 1), similar to the findings in the basic experiments. However, MMP-9 did not emerge as a significant independent predictor in our analysis, nor did it affect the correlation between NGAL and CV events in the multivariate analysis.

NGAL levels play an important role in early detection of AKI. Increase in serum NGAL levels have been reported after coronary angiography and PCI due to contrast material instillation and activation of neutrophils by direct tissue damage [26, 27]. The angiography-induced increase in serum NGAL levels lasted for at most 24 h. In our study, we only recruited post-PCI patients with stable CAD after at least one month of medical treatment and observation, which excluded the confounding effect of PCI on NGAL levels.

In a recent study investigating coronary angiography-confirmed STEMI and stable angina pectoris (SAP), serum NGAL levels were found to be positively correlated with CAD severity and a higher SYNTAX score [16]. Serum NGAL levels are reportedly higher in patients with STEMI patients than in those with SAP, which was higher than in the normal control groups [16, 28]. However, the predictive value of NGAL levels in patients with stable CAD remains inconsistent. Some studies had reported that high serum NGAL levels can predict all-cause mortality in patients with STEMI treated with PCI, independent of eGFR [29, 30]. However, other studies have indicated that plasma NGAL levels cannot predict mortality independently of renal function [31]. Moreover, the relationship between NGAL and CAD risk is non-linear and dependent on systemic inflammation and renal function [32]. Our study detected a linear relationship between serum NGAL levels and several CV outcomes in post-PCI patients with stable CAD even after adequate adjustment of serum creatinine levels. Furthermore, the better predictive value of traditional risk model in addition to serum NGAL levels was also confirmed by NRI and IDI calculations. With regard to the outcomes of AMI and CV death, the significant results in NRI and IDI analyses, but not in the AUC analysis, may be attributed to the limited sensitivity of AUC to small changes, particularly when the baseline risk model (e.g., age, gender, hypertension, diabetes, smoking status, creatinine, LDL) already exhibits high discriminative performance. Theoretically, NRI and IDI are more sensitive to nuanced changes. The statistically significant improvements in NRI and IDI highlight the value of incorporating N-GAL into the traditional risk model, as it may more accurately differentiate individuals at high and low risk. Our findings demonstrate that adding N-GAL to the traditional risk model improves the ability to identify individuals at higher risk for specific cardiovascular outcomes, particularly CHF, MACE, total CV events, revascularization, and new-onset hemodialysis. Studies that found no significant predictive value for NGAL in stable CAD patients reported lower average serum NGAL concentrations than those in studies, including ours, that suggested a stronger predictive role. For observational studies, the different in conclusions might be related to the different NGAL measurement assays, baseline characteristics, or NGAL distributions in the study population. Hence, serum NGAL levels have the potential to be a CV prognostic marker because it is well established that NGAL levels are increased in CHF, ischemic stroke, AMI, and stable CAD. However, more evidence is needed to achieve clinical utility of NGAL as a biomarker to guide those high-risk patients whether to receive more aggressive medical treatment or more frequent examinations.

The main strength of our study was the enrollment of a large number of patients with stable CAD patients, which accounts for a large proportion of the CV outpatient department and was representative of the general population. Additionally, this was a prospective study cohort assembled from different centers in Taiwan. However, some limitations of our study must be addressed. First, there might be a selection bias because the clinical data were collected from medical centers only. Although the criteria for patient enrollment were clearly defined, a selection bias related to patient adherence cannot be excluded. Second, there might be residual confounding factors from unmeasured and unknown covariates that would distort the associations. Nevertheless, the issue of unmeasured confounding factors should not undermine the importance of the study findings. Third, our study may not adequately evaluate the correlation between NGAL and CAD severity. While previous studies have demonstrated this correlation [8, 16], our study categorized vessel involvement simply as SVD, DVD, or TVD across NGAL tertiles and did not reveal the same correlation. One possible explanation is that, even within the same level of vessel involvement, SYNTAX scores can vary significantly, reflecting different severities of CAD. Thus, the categorization of SVD, DVD or TVD may not fully capture CAD severity as precisely as a more detailed measure like the SYNTAX score. This approach limits our study’s ability to assess the relationship between NGAL levels and CAD severity accurately. Nevertheless, we believe our findings may pave the way for further studies to explore the utility of serum NGAL in risk stratification and prognosis prediction in different geographic and racial cohorts.

In conclusion, circulating NGAL levels may be an independent predictor of future CV events in patients with stable CAD who have undergone PCI. Furthermore, it could improve risk stratification of traditional risk factors for secondary prevention of CV events in these patients. Larger prospective studies are warranted to validate whether elevated NGAL concentrations can serve as a promising prognostic biomarker in different populations.

## Data Availability

No datasets were generated or analysed during the current study.

## Abbreviations

ACE: Angiotensin-converting enzyme
ACS: Acute coronary syndrome
AIC: The Akaike Information Criterion
AKI: Acute kidney injury
AMI: Acute myocardial infarction
ARB: Angiotensin receptor blocker
AUC: Area under the curve
BMI: Body mass index
BP: Blood pressure
CAD: Coronary artery disease
CHF: Congestive heart failure
CI: Confidence interval
CV: Cardiovascular
DHP-CCB: Dihydropyridine calcium channel blocker
DVD: Double vessel disease
ECG: Electrocardiography
eGFR: Estimated glomerular filtration rate
ELISA: Enzyme-linked immunosorbent assay
FPG: Fasting plasma glucose
HDL: High-density lipoprotein
HF: Heart failure
HR: Hazard ratio
hsCRP: High-sensitivity C-reactive protein
IDI: Integrated discrimination improvement
IL-6: Interleukin-6
LAD: Left anterior descending artery
LCX: Left circumflex artery
LDL: Low-density lipoprotein
LpPLa2: Lipoprotein-associated phospholipase A2
LVEF: Left ventricular ejection fraction
MACE: Major adverse cardiovascular events
MMP-9: Matrix metallopeptidase-9
NGAL: Neutrophil gelatinase-associated lipocalin
NRI: Net reclassification improvement
NSTEMI: Non-ST-segment elevation myocardial infarction
NT-pro BNP: N-terminal pro-B-type natriuretic peptide
NTBR: National Taiwan Biosignature Research
PCI: Percutaneous coronary intervention
RCA: Right coronary artery
ROC: Receiver operating characteristic
SAP: Stable angina pectoris
STEMI: ST-segment elevation myocardial infarction
SVD: Single vessel disease
TIMP-1: Tissue inhibitor of metalloproteinase-1
TNF-α: Tumor necrosis factor-α
TNFSF14: Tumor necrosis factor superfamily 14
TVD: Triple vessel disease
WBC: White blood count
